# A computer science and robotics integration model for primary school: evaluation of a large-scale in-service K-4 teacher-training program

**DOI:** 10.1007/s10639-020-10355-5

**Published:** 2020-11-03

**Authors:** Laila El-Hamamsy, Frédérique Chessel-Lazzarotto, Barbara Bruno, Didier Roy, Tereza Cahlikova, Morgane Chevalier, Gabriel Parriaux, Jean-Philippe Pellet, Jacques Lanarès, Jessica Dehler Zufferey, Francesco Mondada

**Affiliations:** 1 MOBOTS Group of the BIOROB Laboratory, Ecole Polytechnique Fédérale de Lausanne (EPFL), Lausanne, Switzerland; 2 LEARN – Center for Learning Sciences, Ecole Polytechnique Fédérale de Lausanne (EPFL), Lausanne, Switzerland; 3 Computer-Human Interaction in Learning and Instruction (CHILI) Laboratory, Swiss Federal Institute of Technology (EPFL), Lausanne, Switzerland; 4 Flowers team, INRIA, Université de Bordeaux, Ensta Paris Tech, Bordeaux, France; 5 Swiss Graduate School of Public Administration (IDHEAP), University of Lausanne, Lausanne, Switzerland; 6 University of Teacher Education (Haute Ecole Pédagogique) Vaud, Lausanne, Switzerland

**Keywords:** Computer science education, Professional development, Elementary school curriculum, Computer science unplugged, Educational robotics, Curriculum implementation

## Abstract

Integrating computer science (CS) into school curricula has become a worldwide preoccupation. Therefore, we present a CS and Robotics integration model and its validation through a large-scale pilot study in the administrative region of the Canton Vaud in Switzerland. Approximately 350 primary school teachers followed a mandatory CS continuing professional development program (CPD) of adapted format with a curriculum scaffolded by instruction modality. This included CS Unplugged activities that aim to teach CS concepts without the use of screens, and Robotics Unplugged activities that employed physical robots, without screens, to learn about robotics and CS concepts. Teachers evaluated positively the CPD and their representation of CS improved. Voluntary adoption rates reached 97% during the CPD and 80% the following year. These results combined with the underpinning literature support the generalisability of the model to other contexts.

## Introduction

While no consensus has been reached yet, the dialogue around emerging skills and their role in school curricula has evolved from debating the issue of whether computer science (CS) should be introduced in the standard curriculum, to how its introduction should be done (Webb et al. 2017). In fact, there exists no single, common, or best way of introducing CS in the curriculum and, in spite of the many benefits put forth in the literature, both CS and robotics have had a hard time integrating into classrooms as part of the standard curriculum. The increasing number of initiatives actively working on integrating CS in the curriculum, some even with a focus on robotics (e.g. in France with Calmet et al. 2016), have met many barriers that very often hinder the success of such projects in terms of long-term adoption in classrooms. Moreover, although there are examples of successful national endeavours in terms of CS integration, as in the case of Estonia (HITSA Information Technology Foundation for Education 2018), there is scarce documentation detailing the followed approaches, as few countries evaluate their initiatives and/or communicate their results (Balanskat and Engelhardt 2015).

**Fig. 1:**
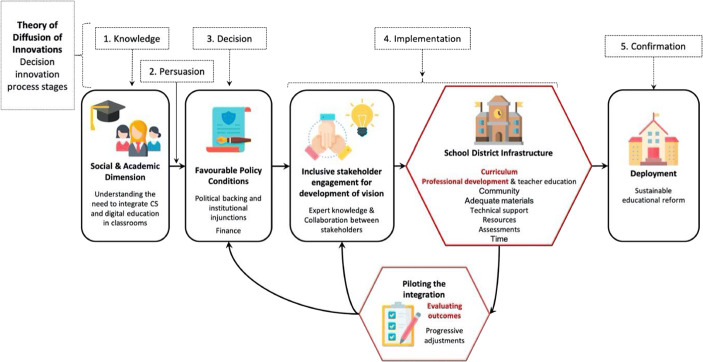
The proposed CS and Robotics Integration Model. The elements in red are considered to be innovative in the context of formal CS and Robotics Education. The model is considered to be complete as 1) the process follows the stages of the Innovation Diffusion Theory (Rogers 2003), 2) the factors cover the barriers recurrently addressed in the literature (Dooley 1999; Kradolfer et al. 2014; Chevalier et al. 2016; Heintz et al. 2016; Webb et al. 2017) the integration was successful. Note that the context from which the model derives is that of a curricular initiative pertaining to CS Education in which robotics was used as one of the means to teach CS concepts. However, we believe that this model applies both in the context of CS Education and Robotics Education, provided the successful adoption of robotics in the pilot program. Illustrations taken from FlatIcon

The current study contributes to filling the afore-discussed void in large-scale analysis of endeavours aiming at introducing CS in primary school curricula by:

1. Presenting the model that was employed to integrate CS and Robotics in the first four grades of primary school (see Fig. 1) and its validation in a pilot program that involved 10 schools (i.e. approximately 350 teachers and 5000 pupils). The model builds upon literature findings (see Section 2.1) to identify key barriers and the strategies we used to overcome them. Particular focus is placed on two elements that are considered key to the success of this project:
  - The format of the CS continuing professional development program (CPD) conceived for K-4 primary school education.
  - The corresponding CS curriculum based on the decomposition into three disciplinary fields that is in line with work by Schiper (2016) and with what was done in other administrative regions^1^: 1) Algorithmics and programming, 2) Information and Data, 3) Machines, Systems and Networks. The curriculum was constructed upon a new categorisation of CS activities based on instructional modality (see Fig. 6). This categorisation ranges from CS unplugged activities, to robotics activities and finally visual programming tasks. In particular, robotics unplugged activities (ones that use physical robots but do not use screens, Rand et al. 2018; Miller et al. 2018) are considered to be a key stepping stone to move beyond CS unplugged tasks and getting one step closer to integrating more advanced CS concepts and environments in the classroom.
2. Reporting the results of the pilot program evaluation, in relation to the above points. Specifically, the analysis revolves around two questions: How did the teachers perceive the training sessions? To what extent did the teachers change their representation of CS (Ketelhut et al. 2020; Bower et al. 2017) and adopt both CS and robotics activities in their classrooms? Indeed, adoption is a key element for any large-scale introduction of a new discipline in the curriculum.

Note that the so-called “trainers” in this paper are the professionals in charge of the continuing professional development (CPD) program. The CPD was decomposed into multiple sessions and that are referred to as “training sessions”.

## Related work

The related work is structured in two parts. Section 2.1 presents the literature that helped construct the model presented in Fig. 1. Section 2.2 then details the related work pertaining to two specific elements of the model: different curricular strategies for CS education in section 2.2.1 and finally examples of professional development approaches that were adopted for CS in other countries in section 2.2.2.

### Constructing a model for CS and robotics educational reform

Implementing changes in the educational system is a complex process with many barriers along the way. These are documented in 1) the literature on diffusion of innovation (Rogers 2003), in 2) the domain of educational technologies (Dooley 1999), as well as in 3) studies detailing what is required for successful education reforms (Schleicher 2018, Ch. 5), 4) studies reporting what was successful and what hindered the integration of CS in the curriculum in other countries (The Royal Society 2012, 2017; Roche 2019), and finally 5) studies reporting recurrent barriers in the literature of CS and Robotics (Kradolfer et al. 2014; Heintz et al. 2016; Webb et al. 2017).

Although the barriers to curricular reform are documented in the literature, no comprehensive model exists that regroups them and that is evaluated in-situ. As such, we present in Fig. 1 the empirical model based on existing generic models of diffusion of innovation but with the specific elements of the introduction of CS as a new discipline in the curriculum. The objective is to provide a complete and comprehensive look into the different elements that are required in order to sustain efficient reform in classrooms. This was then applied in the context of the pilot program of the state-level initiative looking to integrate CS and Robotics into the curriculum. The following sections describe this process for the different components of the model, taking support from the current literature and integrating the contributions from our pilot study.

#### Knowledge, persuasion, decision

Any endeavour begins with the knowledge that a reform is needed and by persuading policy makers to provide *political backing* (Kradolfer et al. 2014) under the form of *institutional injunctions* and *financial support*. Indeed, without the required financial backing and adapted infrastructure, even motivated teachers cannot integrate the CS content in their classrooms (The Royal Society 2012). In the case of our pilot project, a political decision was taken at the end of 2019 by the parliament of the administrative region of the Canton Vaud, ensuring the budget for the pilot and its deployment to all the schools in the region.

#### Implementation

Provided the previous elements are in place it is important to start by *setting the direction through collaboration between all stakeholders* (Kradolfer et al. 2014; Balanskat and Engelhardt 2015; Blikstein 2018; Schleicher 2018). These include all parties with a vested interest in the topic and/or with the expertise required to successfully launch the project: policy makers, those in charge of the continuing professional development (CPD, also referred to as in-service teacher training) and teacher education (pre-service teacher training), experts in CS, unions, teachers and all their hierarchy up to the head of the department of education. Surprisingly, school leaders and teachers in particular are often neglected at this stage, even though they are experts in the field and undoubtedly the ones best suited to reflect and comment on the feasibility of such a project within the classroom (Schleicher 2018, Ch. 5). Furthermore, their knowledge of the terrain and feedback regarding their experience is essential in order to guarantee high self-efficacy. Integrating teachers in the process goes a long way in ensuring high teacher motivation and ultimately increase the likelihood of efficient reform of teacher practices and long-term adoption (Schleicher 2018).

Once a team is in place, the *integration should be piloted with an evaluation of the outcomes* (Schleicher 2018, Ch. 5) in order to provide fast feedback to policy makers and stakeholders to adjust the investments and overall direction. This helps avoid making decisions 1) without considering the constraints of the field and 2) without a direct feedback on both the positive and negative aspects of the identified solutions. Additionally, “experimenting with policy and using pilot projects can help build consensus, allay fears and overcome resistance by evaluating proposed reforms before they are fully introduced” (Schleicher 2018, Ch. 5) which is all the more important in the context of CS in primary school. Surprisingly and sadly, to the best of our knowledge, this was done by just seven countries in Europe (Balanskat and Engelhardt 2015). Moreover, often the concept of a pilot is misunderstood. We consider a pilot in the scientific sense, as a validation of a set of optimal hypotheses based on the current literature, as shown in Fig. 1. The validation is done through evidence-based research standards. To be efficient, this validation loop needs to be done continuously and rapidly, creating a type of scientific interaction between the stakeholders. Many pilots in education are just attempts to introduce new elements, but without a strong validation methodology.

The literature identifies a number of components that should be considered in such pilot initiatives, since they directly influence teachers as well as the school infrastructure and provide useful insight for the deployment.

The *curriculum* (Mubin et al. 2013; Kradolfer et al. 2014; Chevalier et al. 2016) with corresponding *resources* (Sentance and Csizmadia 2017; Benitti 2012; Alimisis 2012) and *assessments methods* (Grover and Pea, 2013) should be provided to the teachers. The lack of a well-defined curriculum was one of the limitations highlighted by The Royal Society (2012) following the first attempt at integrating computational thinking into the curriculum in the UK. In that case, the curriculum was intentionally designed to be broad to avoid constraining teachers but the result was that most students “gain[ed] nothing beyond basic digital literacy skills such as how to use a word-processor or a database” (The Royal Society 2012). This illustrates the need for a well-defined curriculum that provides teachers with the pedagogical content knowledge they require to facilitate the appropriation and adoption of said content (Hubbard 2018). Teachers require having the pedagogical content at their disposal. Indeed, Roche (2019) found that the more the integration of CS was perceived as easy, the more likely teachers were to adopt it. Finally, the question around what students are learning when conducting CS and robotics activities (Strawhacker and Bers 2019) and how to assess the related competencies in a way that can be generalised and scaled to classrooms in K-12 is still being actively researched today (Akram et al. 2020; Grover 2020; Román-González et al. 2019). As such, it is not surprising that teachers, who are not experts in the domain, require guidelines and a methodology that they can use themselves.

In order to facilitate teachers’ adoption of the new curriculum, and the adaptation of their pedagogy, *teacher education and professional development* are widely recognised as key elements (Kradolfer et al. 2014; Mannila et al. 2014; Chevalier et al. 2016; Fluck et al. 2016; Heintz et al. 2016; European Schoolnet Perspective 2017; Webb et al. 2017; Jaipal-Jamani and Angeli 2017). As an example, a lack of CPD and teacher education in CS and CT was documented notably in France and in the UK as a factor leading to low integration in classrooms, in spite of the changes in the mandatory curriculum (The Royal Society 2017; Roche 2019). Teachers not only require the content knowledge (Sentance and Csizmadia, 2017) but also the understanding about how the content should be integrated into the curriculum, “as teachers teach in the manner in which they themselves were taught [and] will likely continue to do so until significant changes in the system itself are effected” (Dooley 1999). In addition to providing resources and content that teachers can integrate in their classrooms, CPD and teacher education must target a change in teacher representation (Ertmer 2005) around CS (Rich et al. 2017), and in this case robotics (Negrini 2020), to avoid negatively impacting adoption (Ni 2009). Beside the fact that teachers may have an inaccurate understanding of what CS is (Israel et al. 2015; Armoni 2011) and may not understand the benefits of integrating the new concepts (Alimisis 2013), teachers tend to believe that the content is too difficult, that they do not have sufficient skills to integrate it into their classroom (low self-efficacy, Chevalier et al. 2016; Sentance and Csizmadia 2017; McGinnis et al. 2020) and that students are already in contact with too much technology (Negrini 2020), to name a few. The result is a direct “influence [on] whether and how they teach concepts of CS” (Israel et al. 2015). As such teachers’ representation of CS and Robotics must be addressed in professional development programs.

Without the necessary *infrastructure and materials* (Schleicher 2018, Ch. 5) even highly motivated teachers would not be able to implement the changes. According to the recent survey by Roche (2019) of 600 teachers in France, 56.2% declared that they did not have the necessary technical infrastructure and resources to implement the curriculum. Similarly, Kradolfer et al. (2014) expressed that the issue was not whether or not teachers found robotics relevant to education, but “the poor capacity of state subsidised schools to adapt to new technological devices”. Additionally, once the necessary infrastructure is in place it is important to consider having adequate technical support (Dooley 1999).

Another constraining element is *time* (Kradolfer et al. 2014; Schleicher 2018), which manifests at multiple levels. Firstly, teachers should not be overloaded with information to avoid “information or innovation overload and burnout” (Dooley 1999). Professional development should thus be conducted over multiple sessions with sufficient spacing between them (Ketelhut et al. 2020; Darling-Hammond et al. 2017). Secondly, integrating the new content requires that they adapt their pedagogy and therefore take time to “absorb information, try ideas out in their classrooms, and then come back for more discussion [as] change frequently fails because insufficient time was allocated” (Dooley 1999). Finally, with “little flexibility in the curriculum to include computing during typical school hours, widespread implementation will typically not occur” (Israel et al. 2015). Indeed, with teachers already struggling to reach the milestones imposed by the curriculum, the addition of CS and Robotics activities are perceived as time consuming, notably since they do not see their immediate utility or their relation to the program. Dedicated time should therefore be allocated so teachers may integrate the content without it being a challenge.

Lastly, a *community* (Brown et al. 2013; Bocconi et al. 2016) is necessary so teachers may exchange practices, activities and experiences as “introspection, collegiality, and a shared sense of purpose or vision combine to create a culture that supports innovation (Staessens 1991)” (Dooley 1999). Indeed, a “survey of over 900 in-service teachers in England concluded that face-to-face events and training, paired with an online community, are considered to be particularly effective in addressing their needs in content knowledge and pedagogical content knowledge related to CT” (Bocconi et al. 2016). But the need for a community is also at the level of the establishment itself with what has been referred to in the literature as “linkers” or change agents (Dooley 1999) and more recently “purveyors” (Israel et al. 2015) whose role is to “act as an interface between the adopters of the innovation and those with a vested interest in seeing the change occur: the stakeholders” (Dooley 1999). Whilst this is often taken up by school principals, our model proposes to have a designated teacher take up that role, following the example of what was done for ICT (Information and Communication Technology) in the Canton Vaud.^2^

#### Confirmation

Deployment should be done progressively to other schools by adapting the approach based on the lessons learnt from the pilot, all the while continuing to monitor and evaluate “periodically after full implementation [as] teachers and school leaders are more likely to accept a policy initiative if they know that they will be able to express their concerns and provide advice on making adjustments” (Schleicher 2018, Ch. 5).

### The CS and robotics integration model

In this paper we cannot evaluate all the elements of the model, and this is not necessary as there is sufficient evidence in the literature to ground them. Therefore, particular focus is placed in this paper on the evaluation of the curriculum and professional development program which are considered innovative in the context of formal CS and Robotics Education (see elements in red in Fig. 1). The literature for these elements is detailed in the following sections.

#### Curriculum design

Open questions concerning CS curriculum design include at what age CS should be integrated, with what content and whether it should be as a standalone discipline or transversal (Barr and Stephenson 2011; Mannila et al. 2014; Yadav et al. 2016; Duncan et al. 2017; Webb et al. 2017). In their review of the status of computing in education in 21 European countries, Balanskat and Engelhardt (2015) highlighted the various means of integrating digital education in the curriculum and found that only 8 countries integrated Digital Education in primary school, and only three as a compulsory discipline. This article presents a model where CS is compulsory and integrated starting primary school (i.e. students aged 4 years old). Introducing the topic early on gives students time to assimilate the concepts without the pressure of major assessments (Thompson et al. 2013), helps reduce pressure and positively affects engagement and diversity (Duncan and Bell 2015), while helping more students be successful (Duncan et al. 2014). The presence of developmentally appropriate CS content for children in preschool (Bers et al. 2014; Sullivan and Bers 2016; Elkin et al. 2018) reinforces this prospect. The hope is that an early CS introduction would lead to increased self-efficacy and also contribute to reducing the gender gap in the field (Webb et al. 2017). Furthermore, many advocate that “the real foundational material that is needed in CS in early years are already typical of curricula” (Webb et al. 2017). Finally, as the focus of early childhood curricula is understanding the natural world, “knowledge of the human-made world, the world of technology and engineering, is also needed for children to understand the environment they live in” (Sullivan and Bers 2016).

Developmentally appropriate CS content for primary school exists under the form of Computer Science Unplugged (CSU), Educational Robotics (ER) and programming activities. CSU activities (Bell and Vahrenhold 2018) benefit from embodied cognition (Romero et al. 2018) without resorting to the use of computers and utilising resources that the teachers in primary school are familiar with. CSU activities are typically short, challenging and often collaborative, as well as less cognitively charged in comparison to programming (Romero et al. 2018). ER activities are inherently interdisciplinary in nature and more and more used as a tool to teach CS concepts Roy et al. 2015; Chevalier et al. 2016; Atmatzidou and Demetriadis 2016; Calmet et al. 2016; Elkin et al. 2018; Chatelin 2019). They are often collaborative and used in a variety of contexts from primary school (Bers et al. 2014; Sullivan and Bers 2016; Athanasiou et al. 2017; Elkin et al. 2018) to tertiary education (Alimisis 2012; Eguchi 2015). ER’s tangible nature helps make “abstract knowledge concrete, [while being more] attractive for children, motivating, and fun” (Chevalier et al. 2016) than the virtual counterparts (Mubin et al. 2013). Indeed, “robots provide an embodiment and the ability to add social interaction to the learning context and hence an advancement on purely software-based learning” (Mubin et al. 2013). Finally, programming activities, both tangible (Bers et al. 2014; Elkin et al. 2018; Mussati et al. 2019) and visual (Resnick et al. 2009; Portelance et al. 2016; Shin et al. 2014) are simplified, developmentally appropriate, and playful environments that help reduce the cognitive load compared to traditional programming environments and render CS notions accessible to a wider demographic. As such, to reach a wider demographic, our model envisions combining CSU, ER and Programming activities in the curriculum. Moreover, we will show the interest of a robotics approach to CSU, called robotics unplugged (RU) which involves the use of a physical robot without the use of screens, making the progression even smoother. The combination of these three components makes it possible for young students to discover core CS concepts both in a scaffolded manner, and with different modalities which should improve their appropriation and generalisation capabilities.

Finally, whether CS should be integrated transversally or as a standalone discipline fundamentally translates into whether the aim is the usage of technology as a tool to enhance learning (i.e. changing the way things are taught without understanding the fundamentals of CS) or whether the aim is understanding core CS concepts as well. We argue that both are important and are part of a larger digital education context which must be considered as a whole. As such, core CS concepts, ICT and questions about digital citizenship should be integrated in order to convey a full picture on the topic. As the curriculum is particularly dense in the proposed model, the decision was taken to design a two-year CPD program. The first year was dedicated to core CS concepts, and the second year to ICT and digital citizenship. Only the results on core CS concepts are considered in the present study.

#### CS continuing professional development approaches

In many cases, CS CPD was not offered by the ministry of education or a central service but “can best be described as a mix of central support coupled with stakeholder-driven initiatives” (Balanskat and Engelhardt 2015). Countries that did not propose a centralised and systematic teacher training program CPD struggled with the integration of CS in the curriculum. This is the case of Australia (Heintz et al. 2016) and New Zealand (Webb et al. 2017; Thompson et al. 2013) that proposed MOOCS and distance courses, or the UK (The Royal Society 2012, 2017) and the US (Heintz et al. 2016) that relied on third-party organisations to create a network and supply reference materials. In France, although trainings were organised locally (Balanskat and Engelhardt 2015), under 20% of teachers reported having followed professional development programs (Balanskat and Engelhardt 2015). The consequence was that only 38.9% of 600 teachers had integrated CS in their classrooms a year into CS having officially entered the curriculum (Roche 2019, p. 159). Of the ten countries reviewed by Heintz et al. (2016), only Estonia had formal professional development courses offered by the centre of IT Education, funded by the Ministry of Education and Research. As of March 2018, their technology program had “affected 85 percent of Estonian schools and 44 percent of kindergartens with its activities in five years” (HITSA Information Technology Foundation for Education 2018). These examples indicate that teacher training should be organised at the national or state level, and not left up to small organisations or initiatives. They should be standardised and compulsory for all teachers, all the while respecting the principle of a pilot with continuous improvement loop proposed in the CS and Robotics Integration Model (see Fig. 1).

To the best of our knowledge, there exists no unique or standard approach to address continuing professional development. As such, the CPD program was conceived following a number of established *training principles* (Ria 2015; Darling-Hammond et al. 2017; Chessel-Lazzarotto 2018). Firstly, the sessions have to be *active and dynamic*, to be close to the type of pedagogy that teachers in primary school are used to having, i.e. learning through kinaesthetic instructional games that are not transmissive but allow students to extract and build their own knowledge from their experiences and projects. Secondly, significant attention was placed on making the CPD *collaborative and in co-construction* (Ria 2015; Darling-Hammond et al. 2017) as “features of organisational climate such as openness, trust, and free communication [are known] to be correlates of innovative behaviour” (Dooley 1999). Indeed, teacher-trainer collaboration was strongly encouraged both to construct a CPD program tailored to the teachers’ needs and to provide an open channel of communication that was shown to play “a key role in overcoming resistance to innovations and in the reduction of uncertainty” (Dooley 1999). Thirdly, this co-construction necessarily implies providing *adapted content and adaptability*. The content has to be developmentally appropriate, easy to transpose to classrooms and to be seamlessly integrated into the curriculum (Dooley 1999) to remove apprehension (Ketelhut et al. 2020 citing Adler and Kim 2018), help teachers feel more confident and promote teacher acceptance (Ketelhut et al. 2020 citing Jaipal-Jamani and Angeli 2017). Additionally, in order to promote collaboration and have tailored adjustments throughout the year, the trainers were required to actively listen, ask for feedback and take the teachers’ opinions into account from one session to the next. Fourthly, teachers have to be *reassured and in confidence*. Confidence is intended both in the trainers, who are experts in CS and pedagogy (Darling-Hammond et al. 2017), as well as in themselves and their capacity to deliver the content in their classrooms, as teachers that are “lacking confidence to integrate an innovation into their instructional program, [...] will tend to ignore it” (Dooley 1999). Teacher self-efficacy is essential both for teacher and student motivation as well as student learning (Zee and Koomen 2016). A lack of confidence therefore can hinder the prospects of long-lasting change, as was the case in New Zealand when they first introduced the new standards (Thompson and Bell 2013). Finally, to ensure durable change and the sustainability of the project, the teachers have to be *accompanied and supported* in the endeavour (Dooley 1999; Darling-Hammond et al. 2017). This support should be provided both in and out of the classrooms, with accompanying personnel explicitly trained for the task (Darling-Hammond et al. 2017) in the schools (here called “personnes ressources” or PRs) as well as technical support, the creation of a community, and the organisation of large-scale events.

In addition to these training principles, the CPD follows the model of sustained duration (Darling-Hammond et al. 2017), with the CS-CPD spanning the course of a year, and an additional year for an ICT and digital citizenship CPD. Furthermore, the CPD was scaffolded for the different levels of primary school with the specific objectives of changing the teachers’ representation around the discipline (Ketelhut et al. 2020) and ensuring sustained adoption.

## Methodology

This chapter describes the pilot program and related evaluation tools. Specifically, based on the principles detailed in section 2.2.2, in the context described in Section 3.1, the CPD pilot program and corresponding curriculum were launched (see Section 3.2). To assess their effectiveness and success, over the course of the pilot a number of evaluation metrics were collected from the participants, as presented in Section 3.3.

### Context

The development and validation of the CS and Robotics integration model for primary school is the result of the research conducted within a political initiative in the administrative region of the Canton Vaud in Switzerland. The initiative, in line with others worldwide, looks *to integrate digital education into the compulsory school curriculum,* with digital education being considered as a whole and including three pillars: CS, information and communication technologies (ICT) and digital citizenship. Therefore, *led by the educational department, and fuelled by the knowledge that students had to acquire the desired twenty-first century skillset, the project* relied on the collaboration and expertise of all relevant institutions:

- The state department of education (DFJC Vaud, Switzerland)
- Professors, computer scientists and roboticists from the local technical university (EPFL, Switzerland)
- Professionals from the local university of teacher education (HEP Vaud, Switzerland)
- University experts in the evaluation of policies and large-scale projects (Unil, Switzerland)
- Public schools from the Canton Vaud.

With reference to the discussion in Section 2.1.2, this initiative seems to unite all the partners required to maximise the chances of success in a unique configuration for Switzerland: political backing and collaboration between the different institutions, public schools and universities.^3^ One particularity of this project which is inherently translational in nature is that teachers are integrated at multiple levels:

- The conception of the content
- The conception of the CPD and delivery of the training sessions
- The accompaniment of teachers in schools (via the so-called purveyors or linkers)
- Testing the content in the classrooms and reporting to the professionals in charge of the CPD program (henceforth referred to as trainers)
- The overall evaluation of the project in collaboration with the researchers.

The collaborators from the different institutions envisioned integrating the three pillars of digital education into the curriculum *starting from the first years of primary school*^4^ (age 4) and to progressively *expand to upper secondary school*. However, to ensure that the endeavour was successful, a pilot initiative with a restricted number of establishments was launched. This provided the opportunity to conduct translational research to evaluate the integration of digital education and proceed to informed adjustments before the regional deployment. The pilot initiative involved 10 schools that were selected from a pool of voluntary establishments. The pilot schools were selected to represent the diversity in the region (i.e. all 92 schools) in terms of location, technical infrastructure, and socio-economics. Teachers from the pilot schools were required to follow a CS professional development program in the first year before progressing to the other pillars of digital education (information and communication technologies and digital citizenship) in the second year. This pool of teachers constitutes the core of the evaluation of the initiative. In particular the evaluation of the CS-CPD and the outcomes in the following year contributes both to validating the CS and robotics integration model, as well as taking informed decisions for the upcoming generalisation and deployment to the entire region.

### Proposed curriculum and CPD program

**Fig. 2:**
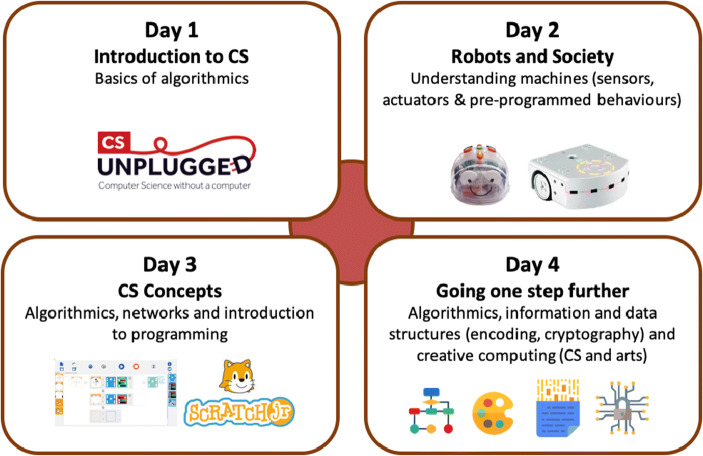
Organisation of the CS CPD (Year 1) which considers CS to be composed of three disciplinary fields (Schiper 2016): 1) Algorithmics and programming, 2) Information and Data, 3) Machines, Systems and Networks. Images taken from the CSU, Blue-bot, Thymio and Scratch Jr. pages as well as FlatIcon

Twelve trainers (seven men, five women) were in charge of the conception of the CPD and the delivery of the training sessions in the establishments. They had complementary backgrounds with varying experience in the domains of teaching, teacher education and CPD, CS and robotics. Each session was led by a pair of trainers, selected to ensure the presence of both pedagogical and technical competencies. This decision was taken to help the teachers feel reassured and in confidence, as dictated by the aforementioned training principles. The devised CS-CPD program was composed of four day-long face-to-face training sessions (totalling 24 hours) spread out over the school year. The CPD was designed to be hands on and interactive, encouraging collaboration between teachers. The sessions included both teacher content as well as student content (also referred to as activities) that were easily transposable to classrooms following the principle of isomorphism (Lebrun 2004 p. 15, Mialaret 1977 p.17) and also presented indications pertaining to classroom management. Throughout the professional development program, 70% of the time was allocated to practical activities that could directly be transferred to classrooms. As detailed in Table 1 and illustrated in Fig. 2, over the course of the days, the teachers were progressively introduced to:

1. CS Unplugged activities, to discover the basics of algorithmics
2. Robotics Unplugged activities (Rand et al. 2018; Miller et al. 2018), to understand the different components of machines (sensors, actuators) and their behaviours by interacting with physical robots and programming them without resorting to the use of screens
3. More advanced CS concepts and visual programming activities
4. Advanced concepts in algorithmics, information and data structures, and finally elements of creative computing (CS and arts).

**Table 1:** Detailed student-centred content that was presented during the CS CPD and conceived to be easily transposable to classrooms. Activity types are presented based on instruction modality and are summarised as CSU for computer science unplugged activities, RU for robotics unplugged activities, RVP for robotics activities involving visual programming and VP for non-robotic activities involving visual programming. The Day refers to the training session in which the content was delivered to the teachers

Activity	Activity Type	Activity Description	Day
The sorting machine	CSU	To understand how computers work and how processes can be parallelised, students do a succession of one to one comparisons and conditional movements on a mat to discover the notion of algorithms	1
The robot game	CSU	A role-playing game where one student plays the computer who has to give the other student, who plays the robot, indications to move along a grid, as a way to discover the notion of language, instructions and programming terms	
The crane	CSU	In groups, the students play the role of the programmer, reader and executer of a code so that the crane (played by the executer) does a specific motion. Notions of bugs and technical choices are also provided.	
The pixel game	CSU	Students learn to transmit a black and white image without showing it and progressively introducing the notion of language, pixels, and encoding.	
Treasure hunt	CSU	Students learn about instruction sequences and conditional statements by moving along a grid.	
Bluebot	RU	Students learn how to translate movements into sequential instructions so that the Bluebot robot moves. These movements can be done with or without a map that can contain obstacles and intermediate waypoints.	2
Pre-Programmed Thymio	RU	Students must first discover the Thymio robot with the different buttons and sensors before being introduced to the pre-programmed behaviours that they must decode and finally use to solve advanced missions.	
Thymio VPL	RVP	Through a visual programming interface, the students learn about event-based programming and use it to solve increasingly complex problems.	
Daily algorithms	CSU	Using flowcharts, the objective of this activity is to show that algorithms are not just specific to computer science but exist in our daily lives (e.g. recipes, grammar, logical problems)	3
Salmon sorting	CSU	A kinaesthetic way to experience to sorting algorithm as a class, the students must line up and go up “the river” one by one and exchange their card with the other students each time they find one which is smaller	
Scratch Jr	VP	This visual programming interface was used for creative and interactive narration so students learn about event based and sequential programming with customisable avatars and environments	
Networks	CSU	A collaborative activity where students simulate the different components of network. They learn about routers, and specifying senders and recipients in order to communicate through networks with different configuration.	
Cryptography	CSU	Introduction to the need to crypt information when it is passed through a network in a way that the recipient be able to decode the message.	4

As anticipated in the introduction, the key goal of the CPD program can be decomposed in two main factors. The first was to ensure positive perception of the CPD format and content. The second was to make sure teachers had a positive representation of the discipline (see Section 4.2.1) and transferred (at least some of) the different activities in their classrooms. This adoption metric is especially significant since the teachers were not obliged to implement the activities in their classrooms.

### Participants and data collection

To evaluate the success of the pilot program along the two axes outlined above, we employed a mixed methods approach. Whenever possible, multiple data sources were triangulated in order to obtain a more reliable view on the matter. Information about the type of data acquired, from which participants, at which point in time, whether it is qualitative of quantitative and how it relates to the different axes of analysis are summarised in Table 2.

**Table 2:** Qualitative and quantitative evaluation methods used in the study and relation to the two axes of analysis

Data Type	Data Source	Questions on	Perception of the CPD	Representation and Adoption	When
Quantitative	Teachers	Representation of CS		X	CS days 1&4
		Evaluation of the CPD	X		CS days 1–4
		Adoption of the CS content		X	CS days 2–4, ICT days 5–6
	Directors	Start of CS CPD teacher related prognostics		X	Aug. 2018
		End of CS CPD director satisfaction	X		June 2019
Qualitative	Teachers	Open questions in the teacher surveys	X	X	CS days 1–4
		End of CS CPD teacher focus group	X	X	CS day 4
	PRs	End of CS CPD PR focus group	X		PR day 4
	Directors	End of CS CPD survey open questions	X		June 2019

#### Teachers

Approximately 350 teachers (98% of which were women, teaching grades 1 to 4 in the 10 pilot public schools, also referred to as the first cycle of primary school), participated in the four days of CS CPD (see Fig. 2) that took place between September 2018 and April 2019. At the end of each session, the teachers were given the pedagogical resources and material required to implement the presented activities in their classrooms. Adoption of the activities in the classrooms was completely voluntary: teachers were free to do all, any or none of the proposed activities (albeit with encouragement to do so if possible).

Following each CS CPD session, the teachers filled out a paper-based questionnaire investigating their perception of the training sessions themselves. The questionnaire items were evaluated on a 4-point Likert scale (min = 1, neutral = 2.5, max = 4). The first day also introduced questions on the representation of CS. Following sessions integrated question on the adoption of the CS activities proposed in the previous sessions. The different items are summarised in Table 3. All data collected in the first year was anonymous, only including the grade taught and establishment. The number of responses obtained per grade and per day are presented in Table 4 where grades 1 and 2 are denoted by 1-2P and correspond to students aged 4–6 years old, and grades 3 and 4 by 3-4P for students aged 6–8 years old. The low response rate in Day 4 is due to survey collection error. Finally, a *focus group* was conducted during the last CS CPD session. Teachers were divided into groups of 10 and were asked to reflect on 1) the content of the training session, 2) the format, 3) how it would be possible to assess the students, 4) perspectives of the impending discipline, 5) their needs 6) representation of the discipline and 7) collaboration within the project. Their responses were collected in the form of post-its (around 1200 overall). The following year teachers electronically responded to adoption surveys to provide information about long-term adoption, as the ICT CPD did not focus on CS activities, nor reprise them. Adoption was not surveyed in Day 5 (Oct. 2019) as this was too early in the school year. The last adoption survey was conducted in March 2020 prior to schools closing due to COVID-19.

**Table 3:** Teacher questionnaire items

Topic	Question	Day
	How do you feel at the end of this day: Reticent?	
Representation	How do you feel at the end of this day: Open?	1
	How do you feel at the end of this day: Confident?	
	The training sessions were rich and interesting	
Perception	The level of difficulty was well adapted	1–4
	The equilibrium between theory and practice was well adapted	
	The appreciation of the content	
Adoption	How many periods did you do per activity?	2–4 & 6–7
	*One period represents approximately 45 min*

**Table 4:** Number of teachers participating in the data collection, by training session day and grade. Grades 1 and 2 are denoted by 1-2P (students aged 4–6 years old), and grades 3 and 4 by 3-4P (students aged 6–8 years old). The category “other” includes teachers that are not from the cycle, specialised teachers, PRs, and teachers for whom the information is missing. In particular in Day 1 many teachers did not provide their grades. Nonetheless, the proportion of teachers in the different grades is coherent with the proportions in later training sessions, notably in Year 2 where the surveys were administered electronically

CPD Sessions Responses	Grades 1-2P	Grades 3-4P	Other	Total
CS - Day 1 (Oct. 2018)	85	96	110	291
CS - Day 2 (Nov. 2018)	130	136	54	320
CS - Day 3 (Mar. 2019)	124	137	45	306
CS - Day 4 (Apr. 2019)	66	111	37	214
ICT (no CS) - Day 5 (Oct. 2019) – No adoption survey	–	–	–	–
ICT (no CS) - Day 6 (Dec. 2019)	146	159	19	324
ICT (no CS) - Day 7 (Mar. 2020)	128	150	20	298
ICT (no CS) - Day 8 (May. 2020) - Cancelled (COVID-19)	–	–	–	–

#### The purveyors of our model: The PRs

Across the 10 pilot schools, 23 voluntary teachers were attributed the role of the purveyors in our model (called “personnes ressources” or PRs here). These teachers followed four days of training sessions in parallel to the others in order to experience the content first hand, acquire additional knowledge and be able *to accompany and support the teachers* of their establishment in implementing the CS activities in their classrooms. Before being delivered to all teachers, each training session was piloted with the PRs, so that they could provide feedback on the content and test the activities in their classrooms before the other teachers. At the end of the CS CPD program, three focus groups of approximately 8 PRs each were organised to evaluate the implementation of CS in their respective schools and understanding what their role in it was. These followed the same procedure as the teachers’ focus groups.

#### School directors

The ten school directors were consulted at the start of the pilot program and following each CPD session. This had two goals: firstly, to get feedback about the progression of the program and, secondly, to collect their views on what was going on in their establishments. In particular, two questionnaires were administered, once at the beginning and the end of the CS-CPD.

## Results and discussion

The following sections report the results of the pilot program evaluation along the axes outlined in the introduction (perception of the CPD, representation of the discipline and adoption of the content) using the tools described in Section 3.

### Perception of the CPD

**Fig. 3:**
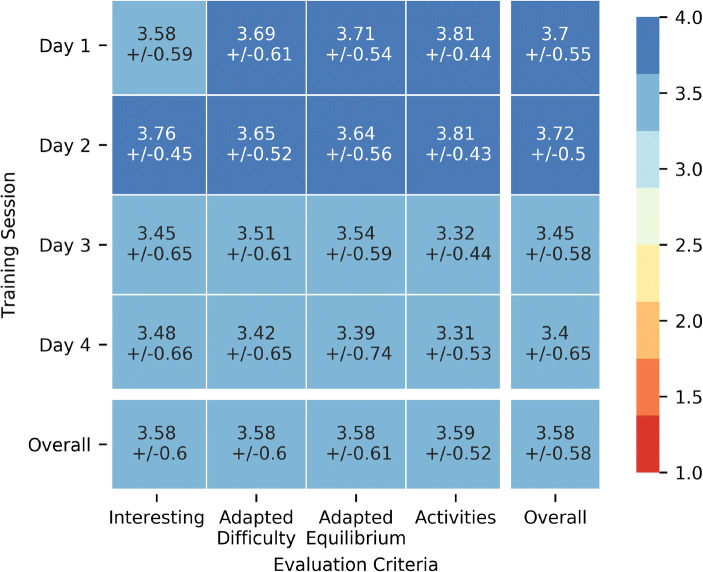
Teachers’ perception feedback on the CPD and their content based on the items in Section 3.3.1

Considering the teacher responses to the four items on perception presented in section 3.3.1, the scores are globally positive with all items exceeding an average of 3.3 on *all days* and the overall satisfaction valuing 3.6 (see Fig. 3). When considering the *open question on what teachers felt was the most useful part of the training session* in *Day 1*, responses indicated that they appreciated the concrete and practical aspects of the training sessions (over 200 responses, *principle: Active and Dynamic*) and the isomorphism that rendered the activities easily transposable to the classrooms (over 70 responses, *principle: Adapted and Adaptable*). Satisfaction is not consistent across days and criteria (Kruskal Wallis *p* < 0.001). Specifically, the CS Unplugged and Robotics Unplugged days (*Days 1 and 2*) were particularly well received (average 3.7) with significantly higher scores compared to other days (*Days 3 and 4*) for adapted difficulty, equilibrium and content (Kruskal Wallis test p < 0.001). In addition, the Robotics day (*Day 2*) significantly differs from all others in terms of interest (Kruskal Wallis test p < 0.001). As the matrix in Fig. 1 reports, it seems that although the teachers generally gave very positive scores (above 3.6 overall, considering all days and criteria), they appreciated less the content related to visual programming and the more advanced CS concepts introduced in *Day 3 and Day 4*. This trend is consistent across the grades that the teachers taught (Kruskal Wallis test between grades intra Day 3 *p* = 0.88 and intra Day 4 *p* = 0.43). Therefore, it would appear that the content (which was the only element that differed throughout the training sessions, see Fig. 2) had a significant impact on the way the training sessions were perceived by the teachers.

A positive reception of the training sessions was not only expressed by the teachers, but also by the school directors. In the final questionnaire administered in June 2019, the directors reported an average satisfaction of 3.9 with the CPD program. In the *open questions* relating to the main successes of the project, they indicated that they believed that there was good support from the teachers for the project, with “a keen interest on the part of the teaching staff” which created a dynamic of collaboration in the various sites (*principle: Accompanied and Supported*). They attribute the quality of the CPD to several elements: the supervision, support during the year and the resources that were rapidly made available to the teachers (*principle: Accompanied and Supported*). At the same time, they put forth elements that might hinder the effectiveness of the deployment to the entire region: logistics of the material, teacher replacement logistics, overloading the teachers, lack of coherence between the different pillars of the program (CS, ICT and digital citizenship) and lack of a comprehensive view of the overall project. Indeed, in terms of teacher overload, the decision was taken not to deliver a differentiated CPD program within the cycle but rather to provide the same content to all. Our rationale was to promote a common CS culture and cohesion throughout the cycle so that teachers may engage in a dynamic of the teams (*principle: Collaborative and in Co-Construction*), confirmed by the data collected during the *teachers’ focus group* that took place on the last day of the CS CPD. Specifically, teachers confirmed the impact of the CPD program in establishing a community of exchange and collaboration between colleagues (36 post-its), whether it be at the level of practices, workshops or equipment (*principle: Accompanied and Supported*).

Finally, although no question about the trainers was explicitly asked over the course of the training sessions, the spontaneous feedback we obtained was positive. In *Day 1*, fifteen *teachers* provided positive comments about the dynamism and competencies of the trainers, with the only negative comment coming from a teacher worrying that trainers might not understand the constraints of students in 1-2P (4–6 years old). This comment however did not appear again in the following questionnaires, nor in the focus groups. Similarly, two out of the three *PR* focus groups highlighted the fact that the trainers had an open attitude and always valued their feedback in the preparation of the teachers’ CPD (*principle: Collaborative and in Co-Construction*). One *school director* also corroborated this by commending the trainers for their attentiveness (*principle: Collaborative and in Co-Construction*) in the *open questions* at the end of the year.

### Teacher representations and adoption

As presented in the Introduction, it is not enough to measure the teachers’ perception of the CPD to evaluate the efficacy of the pilot program. Indeed, our main metric for success is their adoption (i.e. a measure of how much of the content presented in the training ended up being used in classrooms). This metric is especially important in this pilot since the teachers had no obligation whatsoever to conduct any of the activities. However, adoption cannot be disjointed from the teachers’ representation of the discipline, since a negative view is likely to lead to low adoption.

#### Representations

**Fig. 4:**
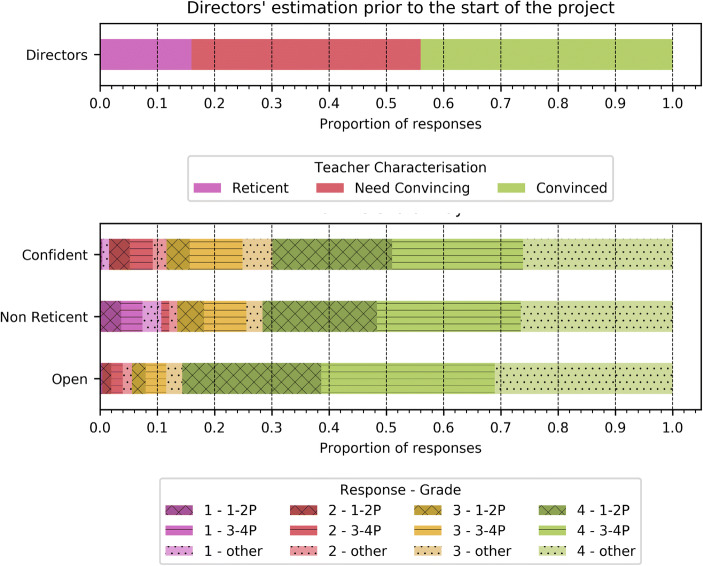
Director estimations compared to the results obtained at the end of day 1 from the teacher questionnaires with respect to reticence, openness and confidence. Negative results are presented in shades of red and progressively go towards positives in shades of green. Note that the reticence question was converted to non-reticence so that responses corresponding to positive outcomes follow the same colour scheme

Initial estimations provided by the *directors* of the establishments in the beginning-of-year questionnaire indicated that 16% of teachers would be reticent, 40% would need convincing and only 44% were already convinced about the integration of CS in their teaching (see Fig. 4). This is coherent with the responses in the open questions provided by the *teachers*: when asked to rate their perception of CS before the start of the CPD program, over 120 teachers (approximately 35%) perceived CS as complex, abstract or had no idea about the subject.

Changes in representation around the discipline are visible already at the end of *Day 1*. As can be seen in Fig. 4, when explicitly asked to evaluate their *reticence, openness and confidence* with respect to the topic, 13% of teachers felt reticent, 94% were open to CS and 88% confident that they could implement it in their classrooms when considering a binary split between positive and negative responses. This does not differ between teachers of different grades (Kruskal Wallis *p* > 0.05 for the three metrics). In the responses provided to the *open question on the evolution of their representation* (263 responses), 58% of teachers stated that their representation evolved positively and 37% that they believed that the discipline was accessible compared to their initial representation of CS (253 responses) that they characterised as “nothing” (22%), “fuzzy” (15%), “complex” (11%) and abstract (6%) with only 12% of positive comments. When explicitly asked about *potential problems in the overall project*, main concerns revolved around the accessibility of the resources (22%), time (21%) and classroom management (13%).

At the end of *Day 2*, in the *open question on how the activities went with the students and what difficulties were encountered*, the 250 teachers having conducted activities at that point reported having highly appreciated the enthusiasm of their students (16%) and facility that the students experienced when conducting the activities (12%). To complement and further support these results, in the final *teachers’ focus group*, out of the 1200 post-its overall (with an average of 40 post-its per sub-topic of the 7 global themes), and although the teachers were not explicitly asked, the teachers again mentioned having a better understanding of CS (13 post-its) and of how computers worked (12 post-its).

We think that a positive representation of the discipline and confidence are crucial ingredients for the adoption of the proposed activities in classroom. As such, an analysis of the adoption of the proposed activities can not only be viewed as a concrete, direct measure of the success of the pilot program, but also as an indirect measure of the impact that the program had on the teachers’ representation of CS. The adoption analysis presented in the following Section spans Year 1, in which the teachers were involved in the CS-CPD, and Year 2, in which CS was no longer the focus of the CPD (see Table 4).

#### Adoption

**Fig. 5:**
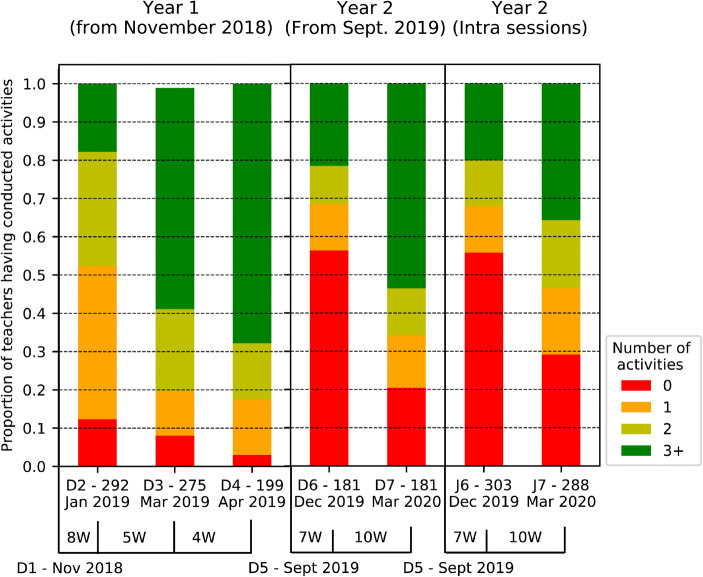
Adoption rates during the CS-CPD (Year 1) and in the following (Year 2). The number of working weeks between sessions is indicated below the graph (and is denoted by a number and the letter W). Adoption is computed only considering the teachers that could conduct activities in their classrooms and based on the number of different activities they implemented (0, 1, 2 or more). The number of teachers considered for the analysis at each session is indicated next to the day. Note that the Year 2 adoption results are shown two ways as adoption was asked relatively to the last data collection (whereas in Year 1 it was asked from the beginning of the year). The first considers only teachers that provided consistent IDs in order to determine the year level adoption, the second considers only the adoption intra-sessions. As adoption results are coherent in day 6, we consider that the teachers that were matched are representative of the overall distribution. Adoption levels were high at the end of Year 1 (92% in March 2019, 97% in April), while in March 2020 it was 80% (for the 181 teachers that could be followed). This is in line with the fact that 12% of teachers in March of Year 1 had conducted only one activity and seems to point towards the novelty effect wearing off

As a first result extracted from the *questionnaire responses acquired in Year 1*, the teachers devoted more than 2300 *periods between November 2018 and March 2019* to proposed activities, in addition to the existing program (see Table 5 and Fig. 7). On average, this represented 0.7 periods per week (with 1 period representing approximately 45 min) for the teachers that conducted the activities by the third training session. This is close to the 1 period per week of CS that was intended by the CPD program in light of the integration of the discipline in the curriculum in the upcoming years. Over the course of *Year 1*, *the proportion of teachers having carried out at least one activity* increased from 88% in November 2018 (out of 357 responses) to 92% in March 2019 (out of 272 responses) and finally 97% in April 2019 (out of 199 responses) when considering only the teacher that could implement the activities in their classrooms (see Fig. 5). That is to say, this excluded teachers that either did not partake in the previous training sessions, were on leave, did not have their own class due to working part time or sharing the classroom with another teacher. In the *comment section of the questionnaire*, of those that did not conduct an activity, only 2, 1 and 0 in days 2, 3 and 4 respectively expressed explicit rejection of the CPD program. This is coherent with the results obtained in the *final focus groups* where only 9 post its out of 1200 expressed an ideological rejection of the project, either due to believing that the priorities lay elsewhere or because they did not believe that CS should be integrated altogether. The *focus groups* provide interesting complementary information to the adoption rates. In spite of the number of periods conducted in the classrooms, many teachers highlighted having not sufficient time at their disposal to implement the activities in their classrooms (25 post-its) and that this was even more complex due to the fact that the material was shared within the establishments, often over several sites (24 post-its). We argue that the matter of time will cease to be an issue once the discipline is officially integrated the curriculum. However, the matter of accompaniment and support is important, notably in the first years of such a program.

**Table 5:** Distribution of the number of periods devoted by teachers to CS activities in their classrooms, based on activity type and grade, considering only the teachers who could conduct activities (i.e., who have their own classroom full time). Between Year 1 and Year 2, the number of CS periods increases by 25%, although the proportion of teachers having conducted the activities decreases, notably for teachers in 3-4P (95% to 79%). Therefore, although less teachers conduct the activities, those who did conducted a much higher number of periods, notably in 1-2P where the number of periods increases between years by 62%

		Year 1		Year 2			
		(275 responses in March 2019^1^)		(324 responses in Dec 2019 6, 298 in March 2020)		(181 responses that can be traced in March 2020^2^)	
		Periods	% of Periods	% of Teachers	Periods	% of Periods	% of Teachers
Activity Type	CS Unplugged (CSU)	1353	59%	85%	1266	44%	61%
	Robotics Unplugged (RU)	926	40%	72%	1426	50%	61%
	Robotics Visual Programming (RVP)	33	2%	7%	57	2%	4%
	Non-Robotic Visual Programming (VP)	0	0%	0%	126	4%	7%
	All	2312	100%	92%	2875	100%	80%
Grades	1-2P	807	35%	88%	1305	45%	84%
	3-4P	1227	53%	95%	1407	49%	79%
	Other	163	12%	91%	124	6%	75%

^1^Although 309 responses were provided in Day 3, 34 teachers could not conduct activities (16 in 1-2P, 5 in 3-4P and 13 others), as such, proportions of teachers are computed based on the remaining 275

^2^As the number of periods in Year 2 were asked in the interval of time from the last training session (compared to the beginning of the year in Year 1), teachers had to consistently provide their IDs. Only 191 did so, and of these 10 could not conduct activities (2 in 1-2P, 5 in 3-4P, 3 others). Proportions of teachers are therefore computed based on the remaining 181.

Even though the PRs’ role was defined as one of accompaniment and support in the schools, the *PRs* reported in their *focus group* that the teachers rarely solicited them for help in the classrooms. The PRs attributed this to high teacher self-efficacy and to the practical, concrete and transferable nature of the activities presented (principle: Adapted and Adaptable). From the teachers’ perspective, in *Day 3*, 75% of 1-2P teachers who conducted an activity did so with half of the class, while teachers in 3-4P did so with the entire class 80% of the time. We infer that there is a need for accompaniment, notably in the first half of the cycle. However, teachers in 1-2P often teach in pairs and therefore do not necessarily require additional assistance.

The adoption results during the CS CPD carry on in the second year of ICT CPD which did not reprise any CS related content. As Fig. 7 shows, teachers in *Year 2* conducted a higher number of *periods* (2875 between September and March 2020, with an average of 1.0 period per week), still in spite of not having any officially allocated time for CS in the curriculum. This suggests that the teachers were able to appropriate the content and gained in self-efficacy. However, when considering the *proportion of teachers having conducted the activities* (see Table 5), it decreases from 92% in *March 2019* to 80% in *March 2020*. We hypothesise that 1) the teachers who did not adopt in Year 1, did not do so also in Year 2 and 2) as a sign of novelty effect wearing off, the portion of teachers that had conducted just one activity (15%) by Day 4, did not do any by Day 7 (see Fig. 5). Additionally, the results from the second year reveal that the lack of adoption was not due to the teachers doing something else related to digital education. Indeed, the teachers that did not do a CS activity between Day 6 and Day 7 did not do an ICT activity either. However, it is possible that a number of teachers had planned to implement one or more activities in the last months of the school year (and couldn’t due to COVID-19 forcing schools to close). Several elements support this hypothesis.

- Adoption levels steadily increased both years. In Year 2, although initial adoption levels were low, the same positive trend was noticed: adoption rates nearly doubled between Day 6 and Day 7 going from 45% to 80% (see Fig. 5).
- When the teachers were asked why they did not adopt in Day 6 (172/320 teachers in December 2019), 29% said they had other priorities, 27% that they did not have time and 19% both. Nonetheless, approximately 50% of the teachers that could be followed between day 6 and 7 went on to conduct activities in their classrooms.
- In the open comments in Day 6, five teachers insisted that the CS content was better adapted to the second portion of the school year. This is corroborated by the fact that 46% of those that said they did not have time and 42% of those that said they had other priorities in Day 6 conducted an activity between Day 6 and Day 7.
- Additionally, 50% of those who mentioned not feeling confident in Day 6 still went on to conduct activities by Day 7 (12/24). These facts suggest that there are other factors at play than time, priorities and confidence when trying to understand why some teachers consistently do not adopt.

**Fig. 6:**
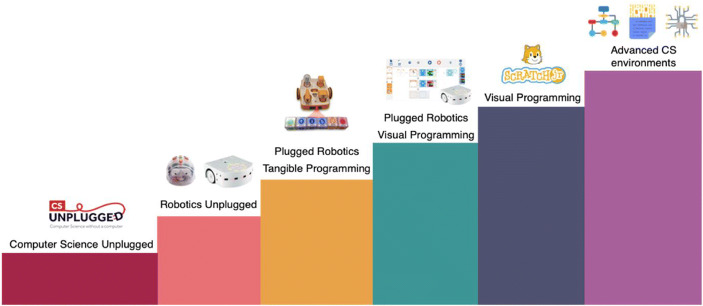
Ideal teacher CPD curriculum progression which 1) proposes to start with CSU activities as they are easy to appropriate and help increase teachers’ confidence and progressively go towards more complex modalities and 2) decomposes robotics activities into robotics unplugged, tangible plugged robotics and visual plugged robotics. The additional tangible layer should help teachers get one step further in their adoption of CS. Images taken from the CSU, Blue-bot, Thymio, Kibo, Thymio VPL and Scratch Jr. pages as well as FlatIcon

**Fig. 7:**
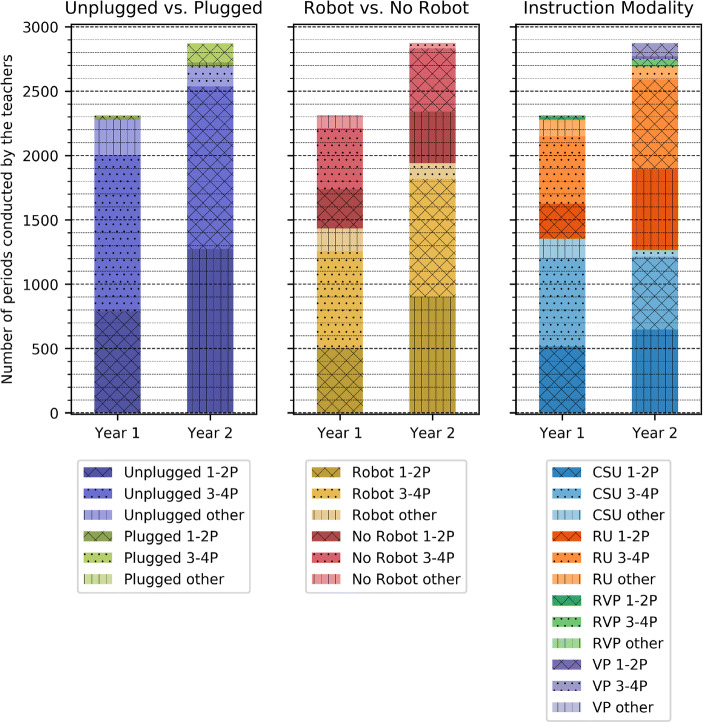
Number of periods conducted by the teachers in Year 1 and Year 2. Adopted activities are the ones proposed in the CPD, and they are the same in the two years. The adoption results are displayed three ways. The first is considering whether the activity was plugged or unplugged (i.e. using a screen or not) whilst the second is based on whether the topic revolved around the robot and understanding how it works or not (some activities in the CSU category, have robotics as a topic). The third column categorises activities based on their instructional modality into CS Unplugged, Robotics Unplugged, Robotics with Visual Programming and Visual Programming activities. The overall number of periods increases between Year 1 and Year 2 with a notable leap in the number of Robotics Unplugged activities, in spite of the logistic difficulties often mentioned by the teachers during the CPD

When considering the *number of periods with respect to the activity type* (see Fig. 7) we observe a predominance of unplugged activities, which is coherent with the content that was proposed. Specifically, 40% of CS activities conducted by teachers were robotics unplugged activities in *Year 1*, compared to 50% in *Year 2*. The limited proportion of plugged activities both in Year 1 and Year 2 is coherent with the reticence regularly expressed towards the introduction of screens in the lower levels of primary school, as well as the satisfaction results which were significantly lower in the third and fourth day of the training sessions. This suggests that introducing CS via CS Unplugged activities, followed by Robotics Unplugged activities can help achieve successful adoption of CS principles in classrooms, regardless of the question of plugged activities. We hypothesise that introducing tangible programming (Mussati et al. 2019) would help climb one step higher up the CS ladder (see Fig. 6), even though it is likely non-essential in the targeted grades.

To that effect, rather than considering dichotomic categories such as unplugged/plugged and robotics/non-robotics related, we employed a new classification of the activities, based on the instructional modality. This considered CS Unplugged activities (CSU), Robotics Unplugged (RU) activities, Robotics activities with Visual Programming (RVP) and Visual Programming activities (VP). It is interesting to observe that the type of activities implemented by teachers in their classrooms evolved towards more robotics unplugged activities in Year 2 (see Table 5 and Fig. 7), notably for teachers in 1-2P.^5^ Additionally, an increase in visual programming tasks can also be observed, even though this is done by a minority of teachers (7% of teachers that can be traced between days), and mainly in 3-4P (79% of VP periods, see Fig. 7). We believe this method of classifying the activities is better than just categorising as plugged versus unplugged, or robotics related versus not. This not only provides a guideline for the increments to be used in the teacher development program, which we believe contributed to the successful adoption, but also helps grasp the nuances of what the teachers do in their classrooms. This characterisation by instructional modality provides interesting insights on the time teachers need to appropriate the content. Indeed, although the teachers appreciated Day 2 the most (robotics day), they conducted more CSU than Robotics Unplugged (RU) activities in Year 1. However, in Year 2, teachers autonomously did the RU activities, and in greater quantity than the CSU ones. Hence, there seems to be a delayed effect for certain activities based on the instructional modality: CSU requires little time to appropriate compared to RU activities, and this is even more true once programming is involved. This confirms the importance of starting with CSU activities as they are easy to appropriate and likely improve teacher self-efficacy. The finding also highlights the importance of having training sessions spread out in time and an analysis which is longitudinal and expands over multiple years.

## Conclusion

This paper presents a model for CS integration in schools and the validation of two of its core elements through a pilot project that was conducted in the Canton Vaud, Switzerland, as a first step in a large-scale initiative to integrate CS into primary school education. Based on our experience and the existing body of literature, we believe that the two core elements that significantly contributed to the success of the project were the format of the training sessions and the curriculum design. Both of these elements impacted strongly the perception of the CPD program by the teachers, their representation of the discipline and finally the adoption of the CS content in their classrooms. Where the curriculum is concerned, the use of educational robotics (under the form of Robotics Unplugged and Robotics Visual Programming activities) bridges the gap between CS unplugged and visual programming tasks. As such it gives young students the possibility of discovering a wider range of CS concepts by exploiting modalities that are developmentally appropriate. Indeed, there is a level of concreteness fading (in the sense of going from “lived” to “perceived” and finally “conceived)”) that occurs as soon as the succession proposed here in the model is implemented. Although the usual way to assess a model would be to conduct a complete assessment of all its different elements, we believe that the results of this pilot program, in addition to the evidence provided by the literature, help validate the proposed model as a whole, which can be used as an example for other initiatives attempting to integrate CS and/or robotics into their curricula.

Indeed, regarding the evaluation that was conducted, changes in teachers’ representation of the discipline as well as the positive reception of the CS CPD in terms of interest, adapted difficulty, adequate equilibrium of theory and practice as well as content, are evident from the start of the program. This manifested in 94% of teachers saying that they were open to integrating CS in their classrooms by the end of the first session, compared to an initial prognostic by directors which estimated that 40% of teachers would need convincing and 16% would be reticent. The change in representation reflects in the high levels of adoption evaluated over two years. Indeed 2312 periods of CS were globally conducted by 92% of the teachers in March of the first year (0.7 periods per week), building up to 97% of adoption in April of that same year. There was little decrease in momentum in the second year in spite of not having had training sessions related to CS concepts: 80% of teachers conducted a total of 2875 periods by March, reaching the targeted 1 period per week of CS in the corresponding classrooms. We attribute the success of the project to having addressed all the barriers evoked in the literature, with in particular an adapted set of training principles and corresponding curriculum content. We also believe that the results should generalise well in the deployment phase as the 10 pilot establishments were selected to be representative of the whole region.

A number of limitations were also identified. Although teachers globally appreciated the training sessions, it was complex for them to see the bigger picture and understand not only how the content was interconnected, but also how it more globally linked to the overall digital education theme, that includes CS, ICT and digital citizenship. As such, the training principles may be lacking an element on clear direction and objectives. From the analysis perspective, the data acquisition method did not help match teachers throughout the training sessions in Year 1. Such information would have been useful to construct teacher profiles and see how the adoption correlated with the feedback that the teachers provided over the course of the CPD program.

Based on the obtained results, notably in terms of adoption, the next step will be to cement the curriculum for the four first years of primary school. Additionally, students were not evaluated, although teachers provided feedback on their classroom experiences. Once the curriculum is set, it will be important to expand the analysis to the students to see what they are learning and make sure that this corresponds to the learning objectives set by those drafting the curriculum.

Finally, for future CPD programs, it would be important to expand on the current analysis framework and gain additional insights into teachers’ representation of CS and evaluate how it evolves throughout the program in relation to their perception of the CPD format and content. There was also insufficient indication regarding teachers’ self-efficacy and how confident they felt in integrating the different content in their classrooms, as this was just asked globally in the first CS training session. It is important to have this information at the level of the different activities as the instructional modality is likely to have an effect on the outcome. Finally, it is important to understand the factors which influence adoption, considering elements such as self-efficacy, interest and perceived utility with respect to the different content presented and not just globally. Indeed, teachers must not only find the content interesting, but also see how it benefits their students, with both elements constituting an indicator of autonomous motivation. In particular, we would like to understand what the reasons for non-adoption are in order to find solutions in the long term to ensure the sustainability of the project.

## Data Availability

The data is available on Zenodo starting from June 30th 2021 (10.5281/zenodo.4081555).

## Footnotes

1 See the case of Zurich here

2 See https://www.pressmitic.ch/ for more information regarding their role in the pre-exisiting ICT curriculum.

3 Including the backing from industry that was recommended by Blikstein (2018) with Apple supporting the endeavour.

4 The initiative concerns all 92 public schools of the a dministrative region of the Canton Vaud. That includes approximately 6000 primary school teachers, 450 secondary school teachers and 98,800 students (Statistique Vaud 2020).

5 Although it would seem that the 1-2P were comparatively less involved in Year 1 (35% of periods, compared to 53% for the 3-4P in first year and compared to 45% in second year). This could be due to the number of teachers classified as “other”, which significantly decreased in Year 2 when we moved to online surveys.
